# Status and gaps of research on respiratory disease pathogens of swine in Africa

**DOI:** 10.1186/s40813-020-0144-7

**Published:** 2020-03-12

**Authors:** P. Oba, B. Wieland, F. N. Mwiine, J. Erume, E. Gertzell, M. Jacobson, M. M. Dione

**Affiliations:** 1 International Livestock Research Institute, P. O. Box 24384, Kampala, Uganda; 2 College of Veterinary Medicine, Animal Resources and Biosecurity, Makerere University, P. O. Box 7062, Kampala, Uganda; 3 National Agricultural Research Organization, Abi Zonal Agricultural Research and Development Institute (Abi ZARDI), P. O. Box 219, Arua, Uganda; 4 International Livestock Research Institute, P.O. Box 5689, Addis Ababa, Ethiopia; 5 Department of Clinical Sciences, Swedish University of Agricultural Sciences (SLU), Box 7054, 750 07 Uppsala, Sweden

**Keywords:** Africa, Pigs, Epidemiology, Respiratory, PPRSv, PCV2, *M. hyopneumoniae*, APP, IAV

## Abstract

Over the last two decades, the pig population in Africa has grown rapidly, reflecting the increased adoption of pig production as an important economic activity. Of all species, pigs are likely to constitute a greater share of the growth in the livestock subsector. However, constraints such as respiratory infectious diseases cause significant economic losses to the pig industry worldwide. Compared to industrialized countries, the occurrence and impacts of respiratory diseases on pig production in Africa is under-documented. Hence, knowledge on prevalence and incidence of economically important swine respiratory pathogens in pigs in Africa is necessary to guide interventions for prevention and control. The purpose of this review was to document the current status of research on five important respiratory pathogens of swine in Africa to inform future research and interventions. The pathogens included were porcine reproductive and respiratory syndrome virus (PPRSv), porcine circovirus 2 (PCV2), *Mycoplasma hyopneumoniae (M. hyopneumoniae), Actinobacillus pleuropneumoniae* (APP*)* and swine influenza A viruses (IAV). For this review, published articles were obtained using Harzing’s *Publish or Perish* software tool from GoogleScholar. Articles were also sourced from PubMed, ScienceDirect, FAO and OIE websites. The terms used for the search were Africa, swine or porcine, respiratory pathogens, *M. hyopneumoniae, APP*, PCV2, PPRSv, IAV, prevention and control. In all, 146 articles found were considered relevant, and upon further screening, only 85 articles were retained for the review. The search was limited to studies published from 2000 to 2019. Of all the studies that documented occurrence of the five respiratory pathogens, most were on IAV (48.4%, *n* = 15), followed by PCV2 (25.8%, *n* = 8), PPRSv (19.4%, *n* = 6), while only one study (3.2%, *n* = 1) reported *APP* and *M. hyopneumoniae*. This review highlights knowledge and information gaps on epidemiologic aspects as well as economic impacts of the various pathogens reported in swine in Africa, which calls for further studies.

## Introduction

Pig production accounts for a large share of growth in the livestock subsector worldwide [1]. The growing global human population creates an increased demand for animal source foods. To meet this demand, pigs are one of the preferred species due to their efficient feed conversion and fast growth rates [1]. Accordingly, there has been a substantial increase in the volume of pig meat produced (38% of the world livestock meat consumed) in the last 20 years [2], often associated with intensification of production and increased movement of pigs between countries.

In Africa, the top three countries in terms of pig population are Nigeria with 7.49 million [3], followed by Uganda, 4.23 million [4] and Malawi, 3.65 million [3]. While pig production offers opportunities for both commercial and smallholder producers, the industry faces several constraints [5, 6]. Transboundary diseases such as African swine fever (ASF) pose a threat to international trade, livelihoods and food security due to high economic impacts. The growing trade with potentially sub clinically infected carrier animals or contaminated vehicles, constitutes a risk of disease spread between countries. Besides ASF, respiratory pathogens such as PPRSv, PCV2, *M. hyopneumoniae, APP* and IAV are likely to play an important role, given experiences from industrialized systems. These diseases account for economic losses [6–8] due to mortalities, reduced growth, poor feed conversion and reproductive performance [9], however their impacts on Africa’s predominantly extensive smallholder pig production systems is unexplored and thus unknown.

### Overview of pig production systems in Africa

In Africa, pigs are kept under three major husbandry systems; 1) traditional extensive or free-range backyard system, typically scavenging low input systems (usually 1–10 pigs), 2) semi-intensive systems, usually confined but with partial scavenging (11–100 pigs) and 3) intensive (> 100 pigs) systems [10, 11]. In general, 65–80% of pigs in Africa are still produced in the traditional extensive, low-input systems [11]. This system is characterized by local or crossbred pigs, with limited or no disease preventive measures. Pig marketing is dominated by poorly organized, informal channels, often associated with market information gaps in many sub-Saharan countries [10, 12, 13]. In most countries, farmers prefer local to exotic breeds due to their relative disease tolerance [14, 15], adaptation to local climatic conditions [16], and higher capacity to utilize poor quality feeds [17]. In Burkina Faso and Senegal, farmers prefer indigenous pig breeds that are well adapted to low input production systems [18, 19]. Roaming of pigs under the free-range smallholder systems facilitates easy spread of infectious agents.

On the other hand, intensive production systems that account for about 20% of the production, are characterized by exotic breeds, higher pig intensity and input investments [11]. However, these systems are reportedly on the decline in some countries e.g. Tanzania, South Africa and Nigeria, due to high costs of feeds and capital investments [11].

In all types of production, management systems vary from farrow-to-weaner, weaner-to-finisher, farrow-to-finisher or mixed systems. Studies report limited access to quality feeds, knowledge and extension services, poor knowledge of farmers about best on-farm practices and biosecurity, as key constraints among smallholder systems [10, 11, 20]. A distinction of production systems is of epidemiological significance for disease occurrence and transmission. In extensive production systems common in Africa, the climatic factors, breeds, husbandry practices and the spectrum of infectious agents are so varied that it is difficult to design effective control and preventive measures.

### Description of key pathogens of economic importance

The five pathogens were selected for review due to their high economic importance reported in other regions of the world [21–24]. Economic loss estimates are only available from intensive production systems in US, Europe and Asia, with little information available for smallholder systems in Africa.

PPRSv is a multifactorial, viral infectious disease of swine with important economic implications described worldwide [25]. The economic effect of PPRSv infections is due to deaths, reduced daily weight gain, feed efficiency and reproductive losses [26].

PCV2 infection in pigs is recognized as a principal cause of post weaning multisystemic wasting (PMWS) syndrome [27, 28]. PMWS is a multi-factorial syndrome [29] characterized by weight loss, labored respiration with coughing and dyspnea, and a dark-colored diarrhea [30, 31]. Clinical expression requires involvement of other agents, such as pathogens of the porcine respiratory disease complex (PRDC), or husbandry and environmental stressors [30, 31]. Economic losses due to PCV2 infections include post-weaning mortality [22], reproductive disorders and poor growth [32]. Co-infection infection with *M. hyopneumoniae* was reported to increase severity of PCV2 lesions and incidence of porcine circovirus (PCVAD) associated disease [31].

IAV outbreaks in pigs are characterized by a sudden onset of high fever, anorexia, huddling, tachypnea and coughing [33]. The disease is caused by swine influenza A viruses, subtyped based on hemagglutinin and neuraminidase proteins. The common subtypes identified in pigs include H1N1, H1N2 and H3N2 [34].

*M. hyopneumoniae* causes swine enzootic pneumonia (EP), a chronic debilitating disease characterized by a mild, dry nonproductive cough [35]. *M. hyopneumoniae* contributes to the PRDC. A study showed that average daily weight gain (ADG) of pigs experimentally inoculated simultaneously with *M. hyopneumoniae* and PCV2 was reduced by 110 g between 63 to 133 days post inoculation and mortality increased by 20% [36]. *M. hyopneumoniae* often occurs as a co-infection with viral or bacterial agents such as PRRSv or *P. multocida*, increasing the likelihood of development of severe disease [37].

APP causes porcine pleuropneumonia, an economically important disease of global distribution. The economic consequences of APP can be severe and are mainly due to deaths, reduced ADG, increased feed conversion ratios, and intervention costs [38]. The main clinical features of acute *APP* infection are depression, fever, anorexia, coughing and dyspnea [38], while the chronic form is characterized by fibrous adherences between the lungs and the pleural cavity, caused by pleuritis and lung abscesses [38]. While the economic impacts of PRRSv [26], PCV2 [22, 32], IAV [34], *M. hyopneumoniae* [23] and APP [38] are widely reported in industrialized systems in US, Europe and Asia, their impacts on pig production and productivity in Africa are poorly understood.

The purpose of this review is to compile existing knowledge on occurrence and distribution of these five important respiratory pathogens of pigs in Africa, and to provide an update on the status of research and knowledge to better target future research on pig health and production.

## Material and methods

### Literature search strategy

Harzing’s Publish or Perish software tool (*ver. 6.34.6288.6798*) was used to search for publications from GoogleScholar database [39]. Articles were also sourced from PubMed, ScienceDirect databases, FAO and OIE websites. The *Preferred Reporting Items for Systematic Review* (PRISMA 2009) guidelines were used to search for articles [40]. From published papers and reports that reported descriptive, analytic studies and other official reports, information on research status, spatial and temporal distribution of the five targeted respiratory pathogens of pigs in Africa was compiled. Full text articles and/or those with abstracts, all published in English were considered for this review.

### Inclusion and exclusion criteria

Based on the reported economic importance for the swine industry, five key swine respiratory disease pathogens were identified: *M. hyopneumoniae,* APP, PCV2, PRRSv and IAV. In the initial screening, the title and abstract of full text articles and/or abstracts displaying the following search terms were considered: Africa, swine or porcine, respiratory pathogens, *M. hyopneumoniae,* APP, PCV2, PRRSv, IAV, economic impact, prevention and control, in combination. Only papers that reported on the presence of swine respiratory pathogens in Africa and that were relevant for the review were retained. The quality criteria used for the selection of articles were based on the study design, the laboratory and data analysis methods. Only articles that reported observational studies, cases and reports from national veterinary services were considered. Only review papers that described epidemiologic characteristics of selected pathogens were retained. The search was limited to papers published from January 2000 to October 2019. All selected articles were manually checked, and duplicates were removed.

## Results

**Fig. 1:**
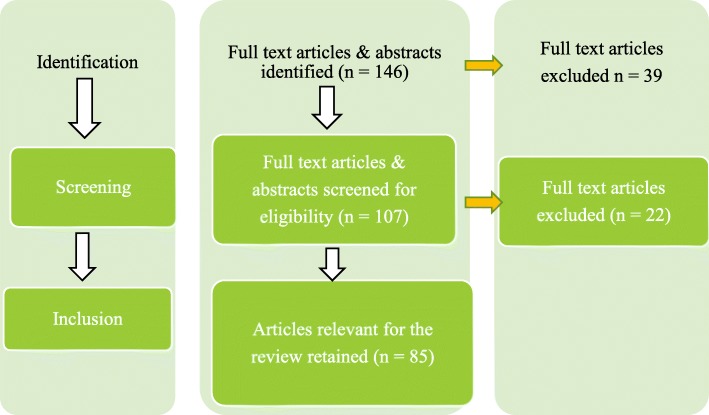
PRISMA flow chart used for the systematic literature review

Altogether, 146 articles relevant for this review were identified. Of the 85 articles retained for this review, seventy-four (87%, *n* = 74) were peer-reviewed scientific publications, five (5.9%, *n* = 5) were from the OIE websites, three (3.5%, *n* = 3) were from FAO website and two (2.3%, *n* = 2) were master/PhD theses, while none were from national veterinary services. Of the 85 papers retained, only 41 (48.2%) studies reported the occurrence of selected respiratory pathogens in swine and of the 41 studies, only 16 (39%) demonstrated the actual occurrence of selected pathogens (immunohistochemistry, HI or PCR), while most studies 25 (61%) were based on serologic assays, suggesting exposure of pigs to these pathogens or closely related strains. Figure 1 below shows a flow chart used for the review.

**Fig. 2:**
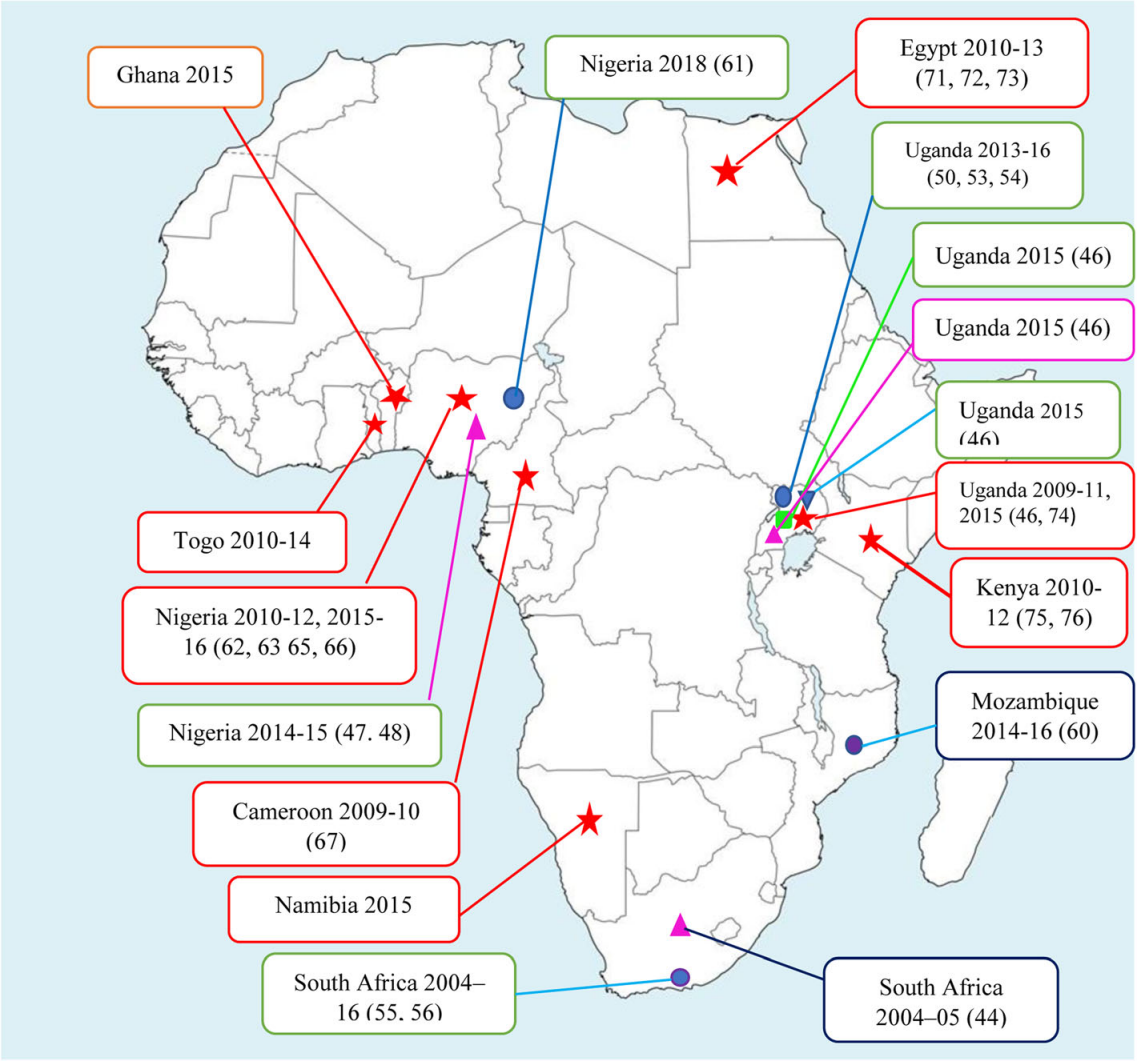
Map of Africa showing the reported occurrence of targeted respiratory pathogens of swine. *Legend: - PCV2 - PRRSv - Swine Influenza A Virus -* APP *and - M. hyopneumoniae*

Figure 2 visualizes locations where the five pathogens of interest were reported.

Of the 41 studies that reported occurrence of targeted respiratory pathogens in Africa, only 63.4% (*n* = 26) were prevalence studies, while the rest (36.6%, *n* = 15) were case studies and molecular epidemiology. The table below presents a summary of the systematic review (Table 1).

**Table 1:** Summary of the prevalence’s and case reports of swine respiratory pathogens in Africa

Pathogen reported	Country	Prevalence (%)	Sample size	Diagnostic method(s)	Year of public.	References
PRRSv	Uganda	1.5	522	ELISA	2018	Dione et al. 2018 [41]
	South Africa South Africa	NA NA	NA NA	- -	2004 2005	OIE, 2004 [42] OIE, 2005 [43]
	Nigeria	53.8	368	ELISA	2018	Aiki-Raji et al. 2018a [44]
	Nigeria	1.4	221	RT-qPCR	2014	Meseko and Oluwayelu, 2014 [45]
PCV2	South Africa	15.9	339	PCR, sequencing	2017 2004	Afolabi et al. 2017b [46] Drew et al. 2004 [47]
	Nigeria	1.4	364	ELISA	2018	Aiki-Raji et al. 2018b [48]
	Uganda	12.0	25	IHC, PCR	2013	Ojok et al. 2013 [49]
	Uganda	77.0	91	RT-PCR	2013	Jonsson, 2013 [50]
	Mozambique	54.0	111	PCR, sequencing	2018	Laisse et al. 2018 [51]
	Uganda	25.0	25	IHC, PCR	2018	Eneku et al. 2018 [52]
	Uganda	4.2	522	ELISA	2018	Dione et al. 2018 [41]
Swine influenza A	Nigeria	8.0	75	ELISA	2015	Adeola et al. 2015 [53]
	Ghana	10.0	50	ELISA	2015	Adeola et al. 2015 [53]
	Togo Ivory Coast Benin	2.5–12.3 0 0	325 498 1112	RT-qPCR RT-PCR RT-PCR	2012 2009–10 2009–10	Ducatez et al. 2016 [54] Couacy-hymann et al. 2012 [55] Couacy-hymann et al. 2012 [55]
	Nigeria	14.0	50	HI	2009	Adeola et al. 2009 [56]
	Uganda Egypt Egypt Egypt	4.9 1.67–4.6 2–4 6–45	522 240 93 -	ELISA HI, RT-PCR HI, ELISA ELISA	2018 2010 2013 2018	Dione et al. 2018 [41] El-Sayed et al. 2010 [57] El-Sayed et al. 2013 [58] Gomaa et al. 2018 [59]
	Uganda	1.4	511	RT-PCR	2014	Kirunda et al., 2014 [60]
	Kenya Kenya Nigeria	16.9 15.9 33.3	759 1084 129	ELISA ELISA RT-qPCR	2015 2018 2018	Munyua, 2015 [61] Munyua et al. 2018 [62] Meseko et al. 2018 [63]
	Cameroon	2.0	104	RT-PCR	2012	Njabo et al. 2012 [64]
	Nigeria	13.7	227	RT-qPCR, HI	2014	Meseko et al., 2014 [65]
*APP*	Uganda	22.8	522	ELISA	2018	Dione et al. 2018 [41]
*M. hyopneumoniae*	Uganda	9.9	522	ELISA	2018	Dione et al. 2018 [41]

Key: *HI* Haemagglutination Inhibition; *IHC* Immunohistochemistry; *RT-PCR* Reverse Transcriptase Polymerase Chain Reaction; *RT-qPCR* Reverse transcriptase real-time PCR; *NA* Not available.

### PRRSv

The first official report of PRRSv was from South Africa in June 2004 when 2407 pigs from 32 farms were slaughtered in the Western Cape province [42]. Two small outbreaks were reported in 2007 from the same area [43]. A recent report suggests that Ugandan pigs were exposed to PRRSv, with an estimated seroprevalence of 1.55% [41]. In West Africa, a serological study in Nigeria found 3 out of 221 (1.45%) samples testing positive for PRRSV antibodies by ELISA [45], while another study in Southwest Nigeria reported a seroprevalence of 53.8% [44]. Accordingly, most African countries to date have never reported outbreaks of PRRSv, its economic impact, or investigated its seroprevalence [66].

### PCV2

The status of PCV2 is unknown in many countries of sub-Saharan Africa [5, 50]. In a study to unravel the transmission patterns of PCV2 at the wildlife-livestock interface in Murchison Falls National Park in Uganda, 91 pigs were sampled and screened for PCV2 antibodies [50]. This study revealed a prevalence of 77% of PCV2b, a genotype associated with PMWS [67, 68]. Other studies in Uganda reported a PCV2 overall seroprevalence of 45.2% (*n* = 236) in Masaka and Lira districts [41] and 25% (*n* = 5) of clinically sick pigs from four districts in central Uganda [52]. A study by Ojok et al. (2013) confirmed the presence of the PCV2 genotype as PCV2b by PCR and IHC [49]. Although limited by sample size (*n* = 35), this study demonstrated the occurrence of PCV2 in Ugandan pigs, as has been shown by others [50].

In the eastern Cape province of South Africa, Afolabi et al., (2017b) [46] reported a prevalence of 15.9% by PCR, with two distinct genogroups (PCV2b and PCV2d) identified by genome sequencing using a Molecular Evolutionary Genetics Analysis (MEGA6 software). In 2001, a study by Drew et al. (2004) confirmed the presence of PCV2 in pigs with clinical signs of PWMS. They concluded that the PCV2 strain found in South African pigs is believed to originate from North America [47]. PCV2d is reportedly a highly infectious genogroup associated with high virulence [69]. The occurrence of two genogroups (PCV2b and PCV2d) in South African pigs suggests a possibility for the emergence of new genotypes by natural recombination, as has been demonstrated to occur between PCV2a and PCV2b viruses [70, 71]. In Southern Mozambique, a recent study aiming to characterize PCV2 genotypes found that PCV2 DNA was detected in 62 out of 111 (54%) samples tested and 23 out of 31 (78%) farms [51]. This study revealed the presence of three PCV2 genotypes (PCV2b 1A/B & PCV2d) and suggested that different PCV2 genotypes circulate in Mozambican pigs. However, the number of pigs sampled in some districts was too low (average 12 pigs per district, range 2–26 pigs) to allow extrapolation to the whole pig population in Mozambique. A higher within-herd prevalence of PCV2 (78%) probably suggests the widespread occurrence of the virus in other swine-producing districts in Mozambique. In Nigeria, a recent serological study revealed a PCV2 prevalence of 1.4% in pigs [48]. For most countries, the status of PCV2 remains unknown, confirming that overall, PCV2 is poorly studied in most of Africa [49]. In the published literature on Africa, no publications were found on the economic impact of PCV2 infection on pig production and the economic losses to the swine industry described in industrialized production systems are difficult to extrapolate to the extensive production systems predominant in Africa.

### Swine influenza a viruses

In West Africa, there were eight cross-sectional studies (ELISAs and RT-PCR) from six countries (Nigeria, Cameroon, Ghana, Cote d’Ivoire, Benin and Togo) with prevalences in pigs ranging from 0.28–44.4% [45, 53–56, 63, 64, 72]. In North Africa, three cross-sectional studies conducted in Egypt reported prevalences ranging from 1.67 to 4.6% by ELISA and HI [57–59]. In East Africa, four cross-sectional studies in Uganda and Kenya reported prevalences (ELISAs and RT-PCR) in pigs ranging from 1.4–8.5% in Uganda and 15.9–17.1% in Kenya [41, 60–62]. In other countries/regions of Africa, the status of IAV is unclear, as no publications were available at the time of this review.

Apart from two serological studies in Ugandan pigs [41], no information was found on the current status of *M. hyopneumoniae* and APP. Further, these studies did not characterize the APP serotypes. To the best of our knowledge, no vaccination is being practiced against *M. hyopneumoniae* or APP in Uganda, which suggests likely exposure to these pathogens. Other than this, no other study documented the *M. hyopneumoniae* and APP occurrence or distribution anywhere in Africa. Thus, further investigations are warranted [41].

### Prevention and control options for respiratory diseases of pigs in Africa

Outside Africa, more research has focused on the development of diagnostic tools and vaccines for prevention of swine respiratory diseases. For PRRSV and PCV2, inactivated and attenuated vaccines are available [31, 73, 74]. Approval for commercial applications, however, is still limited to the US, Europe, China and some Asian countries. Vaccines and therapeutic drugs for the treatment of *M. hyopneumoniae* and *APP* infections are available [23, 75], but from this review, these products were not found to be in use in Africa. While PCV2 vaccination was reported in South African commercial farms [76], the vaccine types were not described. In Gauteng province, South Africa, only 19% of smallholder farmers vaccinate their pigs [77]. In most of Africa, the use of vaccination is constrained by lack of access to vaccines, high costs of delivery, as well as limited cold chain facilities. Despite being the major pig producers, information on current status of control and prevention against the targeted pathogens in Nigeria, Uganda, Malawi and South Africa was not available. In general, knowledge gaps exist on the identity of circulating genotypes/strains, for which specific vaccine types can be targeted. While diagnostic laboratories and national veterinary authorities exist in all countries, no official reports on any of the 5 targeted pathogens were found. Our search was limited to online published reports and may have omitted unpublished works or those in other languages that could have reported on the selected pathogens.

## Discussion

This review compiled research on the occurrence and distribution of five swine respiratory pathogens in Africa. The review only included studies published in English and accessible scientific journals online and may thus have missed papers in French or other languages and did not include “grey” literature. The studies retrieved were mainly undertaken in Nigeria, Egypt, South Africa and Uganda. Of the targeted pathogens, most studies focused on IAV, followed by PCV2 and PRRSv. Serological evidence of *M. hyopneumoniae* and APP were only reported from Uganda. The focus on swine IAV highlights its importance for public health. However, the distribution, genetic diversity, as well as the economic impacts of these pathogens is largely unknown, emphasizing the paucity of data and information.

In addition, sample sizes used in most studies may be insufficient to extrapolate findings at national levels. And several studies focused on a small number of provinces or districts in Nigeria [44, 48, 72] and Uganda [41]. Another important issue is the lack of multi-pathogen surveys, with only one study addressing several diseases [41]. As shown by Dione et al. (2018), co-infections are however common, and up to 68.9% (*n* = 162) of the pigs studied in the Lira and 51.9% (*n* = 149) in the Masaka districts of Uganda tested positive for at least two pathogens. Multi-pathogen surveys are also important in attempts to estimate the burden of disease or to assess the impact of disease complexes such as PRDC.

In contrast with intensive farms, in free-range systems, breeding is generally uncontrolled and haphazardly done [78], as most farmers rely on own stock or hire a boar from neighbors [14, 79], increasing the risk of pathogen spread due to contacts between pigs of varying and unknown health status [7]. In free-range subsistence systems, roaming of pigs is a common practice [10]. In this system, the contact rates between pigs of different herds is high, and given their varied health status, the risk of pathogen spread between potentially infected and susceptible pigs is increased. This may explain the high disease incidence of transboundary diseases [11, 21, 80].

*M. hyopneumoniae* and APP have hardly been studied in Africa, and thus it is likely that their role for swine health and productivity is underestimated. Epidemiological databases on the distribution of APP serovars, Apx toxins, as well as approved diagnostic protocols are thus urgently needed [81]. With respect to swine influenza viruses, scarcity of knowledge on circulating viral subtypes, and their spatial and temporal distribution calls for further epidemiologic studies to guide prevention and control [59, 65]. Most of the IAV studies were conducted in response to the swine influenza pandemic in 2009 and most likely were largely driven by public health risk concerns. Studies on IAV suggest that close linkages at the human-swine-bird interface in West Africa may have implications for continuous virus circulation and possible reassortment of human, swine and avian IAV subtypes, which justifies enhanced surveillance efforts [56].

Beside vaccination, biosecurity measures remain the best methods for prevention of pathogen entry into a herd, including respiratory pathogens [21]. Importantly, the success of vaccination requires evaluation of technical and socio-economic aspects in the context of local production systems. The lack of data limits any attempts to estimate the economic losses caused by these diseases. In general, swine diseases are not considered a priority for surveillance in Africa, which hampers the estimation of their contribution to losses at national level and in turn does not provide evidence for need of more investment in swine health. In contrast, a lot of research and surveillance efforts have focused on ASF due to its high mortality and absence of a vaccine.

Another important fact to keep in mind is that low-input pig keeping systems are prone to high parasite burden which complicates pneumonia diagnosis in pigs due to co-infections. Gastro-intestinal (GIT) nematode infections in pigs cause damage to lung tissues by their migratory larvae*,* causing verminous pneumonia. In this way, they increase their susceptibility to other respiratory infections and may also exacerbate disease severity [82]. This exerts a negative effect on growth and consequently economic performance of herds. GIT nematode infestations are common in African pigs, especially in free-range systems, in which high prevalences (of 30–80%) were reported in various countries [83–86]. Of clinical significance to respiratory disease is *Metastrongylus spp,* whose adult worms can be found in the bronchi and bronchioles. Heavy infestations complicated with bacterial infections is associated with coughing, “thumping” and reduced weight gain [82] . However, in all studies of GIT nematode infections done in Africa, no study documented association between GIT burden and incidence of microbial respiratory infections, despite evidence of associations reported elsewhere.

### Conclusions and recommendations

This review highlights critical research gaps on economically important respiratory pathogens of pigs in Africa, to an extent that makes it impossible to estimate their impacts and evidence for the design of interventions. Most studies focused on IAV, followed by PCV2 and PPRSv. This shows that limited research has been conducted in Africa on the targeted respiratory pathogens, accounting for the lack of data and information. No study on the economic impact of any of these pathogens on swine productivity in Africa was found. Despite the high prevalence of GIT nematode infections reported in pigs in various countries, no studies were found in Africa that determined their association with any of the reviewed respiratory pathogens or quantified their economic impacts. Numerous studies elsewhere reveal economic losses due to nematode infections in swine are substantial [82]. There is need to conduct research on the impact of co-infections of GIT helminths with respiratory diseases in pigs.

The lack of official reports from national veterinary authorities suggests that surveillance systems specific for the reviewed respiratory pathogens in all African countries are either weak or non-existent. Given the largely subsistence structure of pig production, this situation could allow these pathogens to establish in swine populations, which could portend devastating consequences for the pig industry in the continent. Most national surveillance systems focus on single diseases, such as ASF, instead of undertaking a more holistic approach that would allow to gauge the breadth of pig diseases and their impact and thus providing better insights to target interventions. Due to limited investments in animal health, there is need to focus attention to control such diseases that affect productivity, as they threaten the livelihoods of millions of people across Africa.

## Data Availability

Not applicable.
